# Effect of Aggregate Size on Strength Characteristics of High Strength Lightweight Concrete

**DOI:** 10.3390/ma13061314

**Published:** 2020-03-13

**Authors:** Hui Wei, Yang Liu, Tao Wu, Xi Liu

**Affiliations:** School of Civil Engineering, Chang’an University, Xi’an 710061, China; weihuichd@163.com (H.W.); 13891705608@163.com (Y.L.); xliu1205@126.com (X.L.)

**Keywords:** lightweight concrete, aggregate size, mechanical properties, strength prediction model

## Abstract

Effects of aggregate size on the mechanical properties of lightweight concrete (LC) were investigated. Four gradings of lightweight aggregate (LWA) were designed and used to prepare the specimens for compressive strength, splitting tensile strength, and flexural strength tests. An estimating method for compressive strength of LC was then established. The compressive strength of tested LC was up to 95 MPa at 90-day curing time. The test results suggested that the absence of medium-size particles decreased the compaction of LC, therefore the density and compressive strength were negatively affected. Specimens having single size of aggregate showed lower splitting tensile and flexural strengths than that having three sizes of LWA. The parameters of the estimating model were determined according to the test results, and the compressive strength predictions of estimation model were compared with the results from other literature.

## 1. Introduction

Lightweight concrete (LC) has been progressively used in structures such as large-span bridges, high-rise buildings and offshore oil platforms, owing to its superior characteristics in density, specific strength and durability [1,2]. Partially or entirely replacing normal weight concrete (NWC) with LC produces considerable benefits by reducing the dead load of structures [3,4]. Importantly, lightweight aggregate (LWA) is crucial for the properties of LC, since the main reason for the lightweight feature of LC was the light and porous LWA which differs from the denser structures of aggregates in NWC.

In addition to the filling role that aggregates play, the mechanical frame of concrete is also affected by aggregates [5]. It is well known that the grading of aggregates in concrete affects the mechanical properties of NWC [6,7] in terms of the compaction and bond properties of concrete. One of main differences between LC and NWC is the ruptured LWA when subjected to tensile stress [8], which means the fracture path in LC is different from that in NWC. Hence, the different aggregate size of LC could represent a different path of fracture, and then the properties of LC can be influenced. Moreover, several basic properties of LC were related to aggregate size. To date, the gradation of LWA has been proven to have significant influences on the quality of LC [9]. Alengaram et al. [10] investigated the effects of aggregate size and grading proportion of palm kernel shell (PKS) on the strength properties of LC. It was reported that PKS having sizes of 0–5 mm and 10–15 mm influenced the mechanical properties of LC, including compressive strength, splitting tensile strength and flexural strength. Karamloo et al. [11] found that the increasing maximum aggregate size positively affected the fracture toughness and energy of LC. The expanded clay was used as LWA by Nahhab and Ketab to study the maximum size of aggregates on the properties of self-compacting LC [12]. 

It was concluded that the increasing maximum aggregate size led to a decreased superplasticizer (SP) dosage requirement of 700–750 mm slump flow, and maximum size of 10 mm showed best enhancement on the compressive and flexural strengths [12]. However, different types of LWA have different impacts on the mechanical properties of LC. The results from LWAs with low quality, such as PKS, may be not adapted to LWAs with high quality, such as expanded shale. Therefore, relevant research should be more specific.

The influence of LWA on the properties of LC is related to the interfacial transition zone (ITZ) apart from the inherent porous structure and lower strength. A prominent phenomenon, known as ‘internal curing’, substantially influences the structure of ITZ between LWA and the mortar matrix, while this change in the properties of ITZ enhances the bond performance of LWA. However, it was also suggested that the size of LWA affected the bond behavior in LC due to the effect of a varying specific surface [12]. It is difficult to precisely reflect the effect of ITZ on the properties of concrete to date, and the influence of aggregate size on the properties of concrete is also hard to be quantitatively investigated at the microscale.

Therefore, the study of the effect of aggregate size on the mechanical properties of the LC is helpful to comprehensively control the structural performance of the LC. To this end, four groups of specimens having different sizes of LWAs were prepared to investigate the effect of aggregate size on the strength characteristics of LC. The compressive strength, splitting tensile strength and flexural strength of four groups of LCs were analyzed, and then, a simple estimation model for compressive strength considering effect of aggregate size was developed.

## 2. Methodology

### 2.1. Materials

#### 2.1.1. Aggregates

The coarse LWA used in this work is grade 900 expanded shale with density, water absorption and cylinder compressive strength of 1512 kg/m^3^, 2.2 % and 6.9 MPa, respectively. The fine aggregate used is normal weight medium sands with a bulk density of 1510 kg/m^3^.

#### 2.1.2. Cement and Admixtures

Ordinary Portland cement 42.5 [13] was also used for all the mixes, and its chemical composition and physical properties are summarized in Table 1. Additionally, the grade Ⅰ fly ash (FA) and silica fume (SF, produced by Elkem, Shanghai, China, 925U) were used as mineral admixture. Furthermore, BKS-199 polycarboxylic acid superplasticizer (SP, produced by Bok chemicals, Shandong, China), was used to produce the specimens.

#### 2.1.3. Gradation

To investigate the effect of aggregate size on the strength characteristics of LC, coarse lightweight aggregates were divided into three different size ranges: smaller than 4.75 mm, 4.75 to 9.5 mm and 9.5 to 16 mm. The aggregates in each range were applied for one type of concrete mixture; then, aggregates from the three ranges were combined to obtain a combination of gradation. The specific data for the four gradings are given in Table 2.

#### 2.1.4. Mix Proportions

In order to assess strength properties of high strength LC, the ratio of water to binder (W/B) content was taken as 0.27. To keep the only variable in the tests, i.e. the size of the aggregates, one mixture of LC was designed. The concrete mixture was designed according the volume of each raw material, while the volume fraction of LWA was kept as 40%, and the ratio of fine-to-all aggregates is 0.4, which means the sands content was adjusted when the aggregate size was changed. Owing to the lower water absorption of LWAs (2.2%), this was not taken into consideration in the design of the concrete mixture. Furthermore, due to the fact that the mixture was obtained according to the volumetric design method, the use of materials except LWA can be determined at once, while the mass of LWA was changed according to the particle density of LWAs with different sizes. Contents of materials except LWA and sands in the mixture are given in Table 3. Note that despite the aggregates passing the 4.75 mm sieve being definable as the fine LWA, considering the purpose of present study, they were still categorized into the coarse LWA.

#### 2.1.5. Designation of Specimens

Four groups of specimens were prepared and labeled as LC-Ⅰ, LC-Ⅱ, LC-Ⅲ and LC-Ⅳ, corresponding to the four gradings in Table 2. Labels of specific uses in each group, such as compression test, splitting test and flexural test, were not distinguished.

### 2.2. Specimen Preparation

A series of experimental programs was conducted to determine the correlation between the aggregate size and the strength properties of LC, which includes compressive strength, splitting tensile strength and flexural strength tests. For specimens of each group, fifteen 100 mm × 100 mm × 100 mm concrete cubes for the 3-day, 7-day, 28-day, 90-day and 120-day compressive strength tests (three cubes for one curing period), another three 100 mm × 100 mm × 100 mm cubes for the 28-day splitting tensile strength test, and three 100 mm × 100 mm × 100 mm prisms for the 28-day flexural strength test, were cast.

The concretes were mixed by a double-axis mixer (produced by Fangtai, Henan, China) under laboratory-conditions. The LWA was presoaked [14]. Then, cementitious materials, sands and 3/4 of the total water were poured into the mixer. After 1–2 min mixing, the LWAs and the remaining water were added to the mix.

Subsequently, all specimens were cured, demolding was performed at 24 h, and the specimens were placed into a standard curing room maintained at 20 ± 2 °C and 95% ± 10% relative humidity until testing time.

### 2.3. Testing Methods

The oven-dried density, compressive strength, splitting tensile strength and flexural strength of four groups of LC were tested in this study. The test items and the dimensions of related specimens are summarized in Table 4.

#### 2.3.1. Oven-Dried Density

The density of oven-dried concrete was measured by using 100 mm × 100 mm × 100 mm specimens for 28-day compressive strength according to the relevant Chinese standard (JGJ51-2002) [15].

#### 2.3.2. Compressive Strength

The compressive strengths of each group were determined at ages of 3 days, 7 days, 28 days, 90 days and 120 days by following GB/T 50081 [16]. A load-control procedure was applied during the cubic compression strength test with a rate of 10 kN/s. The compression tests were conducted with an electric-hydraulic servo universal test machine having a maximum load capacity of 1000 kN.

#### 2.3.3. Splitting Tensile and Flexural Strength

The American Society for Testing and Materials (ASTM) C78/C78M-18 [17] was followed to evaluate the 28 days flexural strength, and the splitting tensile strengths of four groups of specimens were obtained according to GB/T 50081 [16].

## 3. Results and Discussion

The results of the density (*ρ_d_*), compressive strength (*f_cu_*), splitting tensile strength (*f_st_*) and flexural strength (*f_fl_*) are given in Table 5.

### 3.1. Density

The oven-dried densities of tested specimens were lower than 1950 kg/m^3^, and the mixtures containing the smallest size LWAs (LC-Ⅰ) showed the lowest oven-dried density of all of the tested specimens. Moreover, the concretes labeled with LC60-Ⅲ gave rise to the second lowest density. This phenomenon was also observed by Alengaram et al. [10], and it was attributed to the absence of medium-sized particles, leading to a poor compaction of fresh concrete, which can be confirmed by comparatively higher densities of LC-Ⅱand LC-Ⅳ.

### 3.2. Crack Pattern

**Figure 1:**
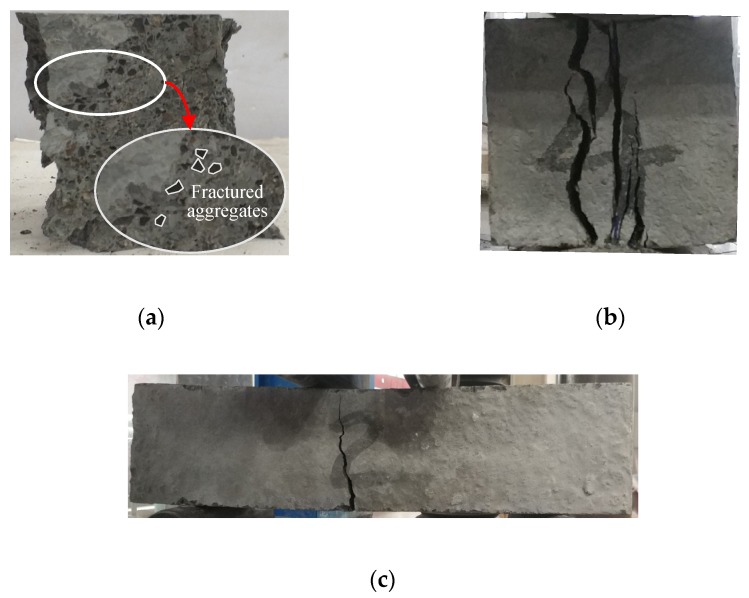
Failures of specimens for compression, splitting tensile and flexural tests-(**a**) typical failure of compressive strength tests; (**b**) typical failure of specimens in splitting tensile test; (**c**) typical failure of specimens in flexural test.

Compared with NWC, the low-strength LWA is the weakest phase in LC, therefore, the crack propagation was considered to appear straight across the LWA in general. A similar cracking pattern was observed in the present study, as shown in Figure 1a, from which numbers of fractured LWAs can be seen. Furthermore, the change in aggregate size hardly affected the crack pattern, considering that fractured LWA was found in all specimens. For splitting tensile and flexural tests, all specimens exhibited brittle failure. The alteration of aggregate size seems not to have had any effect on the failure mode of the LC, since the splitting and flexural cracks of specimens owning different aggregate sizes are visually the same. The typical failures of specimens subjected to splitting tensile and flexural tests are given in Figure 1b,c.

### 3.3. Compressive Strength

**Figure 2:**
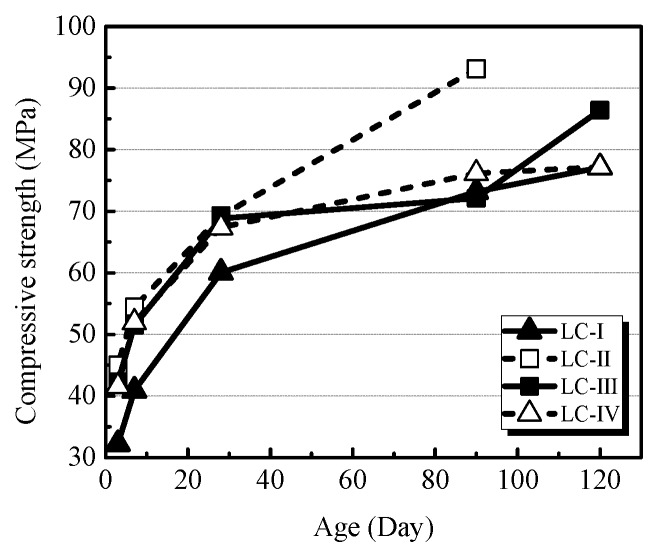
The development of compressive strength on average.

Figure 2 shows the development of the compressive strengths of four groups of LC. The highest compressive strength obtained was up to 95.54 MPa, and all specimens exhibited strength higher than 67 MPa at the 120-day curing time. Similar to the variation of density, LC-Ⅰshowed the lowest initial compressive strength at the curing age of 3 days, the average of which is 22.5% lower than that of LC-Ⅳ (showing the second lowest compressive strength), while the other three groups had comparable 3-day compressive strengths. Moreover, specimens of LC-Ⅱ had the highest compressive strength during the whole curing period and showed the largest strength increment (34.4%) during the 28-to-90-day curing period. It is a general truth that the lower size of particles is related to a greater specific area of surface under certain volumetric conditions [12], and as a consequence, more content of cement is needed to coat the surface of the aggregate. Considering the lower water absorption of used LWA, a similar phenomenon can be obtained in the LC cast in our present study, such that the confinement provided by ITZ on the weak LWA is more obvious, which means that the strength of LWA in that case can be improved. However, more coating cement also undermines the workability of fresh concrete, meaning that the compaction could be relatively poor, and therefore, specimens of LC-Ⅱ showed the highest compressive strength compared with the specimens of LC-Ⅰ and LC-Ⅲ, and accordingly, these specimens of LC-Ⅰ had the lowest compressive strength regardless of the absence of larger aggregates.

### 3.4. Splitting Tensile Strength

The splitting tensile strength of LC was obtained in accordance to the Chinese code (GB/T 50081) [16], and the calculation was written as

(1) $$f_{st}=0.637\frac{F}{A}$$

where

*F* is the force at the failure of specimen, in N;
*A* is the area of splitting surface, in mm^2^.

**Figure 3:**
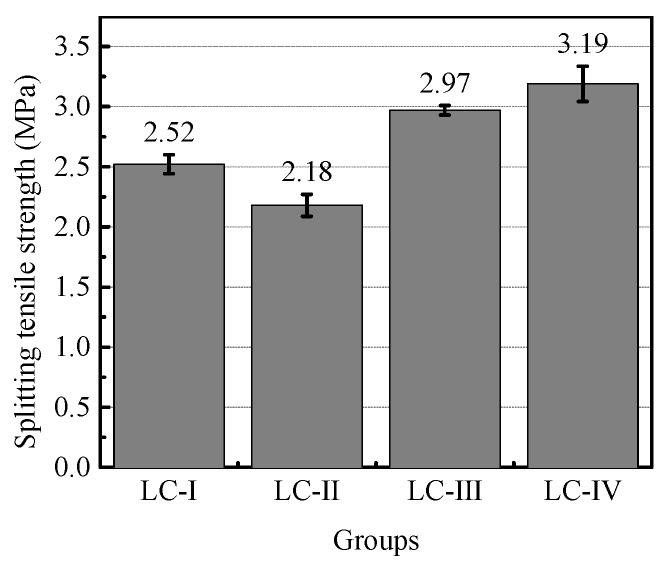
Splitting tensile strength of specimens in four groups.

Figure 3 displays the splitting tensile strength of each group on average. Contrary to the case of compressive strength, it can be seen that LC-Ⅱ owned lowest average *f_st_*, and LC-Ⅳ showed the highest ones. Accordingly, the conclusion can be made that the absence of large and small sizes of LWA decreased the splitting tensile strength of LC, and the greatest difference of splitting tensile strength between LC-Ⅱ and LC-Ⅳ reached 36.7% of the higher one. Furthermore, a continuous grading, such as LC-IV, seemed to be beneficial for the splitting tensile strength of LC.

The splitting tensile strength is governed by both the strength of LWA and the bond between LWA and the mortar matrix [10], therefore, the test results somewhat revealed that the larger size of coarse LWA produced a better bond performance between LWA and the mortar matrix, considering the higher strength of LWA having smaller size [12].

Consequently, the reason for the higher splitting tensile strength can be derived that the combination of three sizes of aggregates generated better bond performance between LWA and the mortar matrix to compensate for the lower strength of each size of LWA. Moreover, it is known that the optimum gradation of aggregates contributes to a denser concrete mix, which has a significant influence on the fracture behavior of concrete [7,18]. On the basis of the denser characteristic discussed in Section 3.1, the greatest splitting tensile strength of LC-Ⅳ was considered to be also improved by the better structure of LC on the microscale.

### 3.5. Flexural Strength

The flexural strength of LC was calculated by the failure force, span and section size of specimens, which can be expressed as:

(2) $$f_{fl}=\frac{Fl}{bh^{2}}$$

where

*F* is the force at the failure of the specimen, in N;
*l* is the span length between two supports, in mm;
*b* is the width of the specimen, in mm;
*h* is the height of the specimen, in mm.

**Figure 4:**
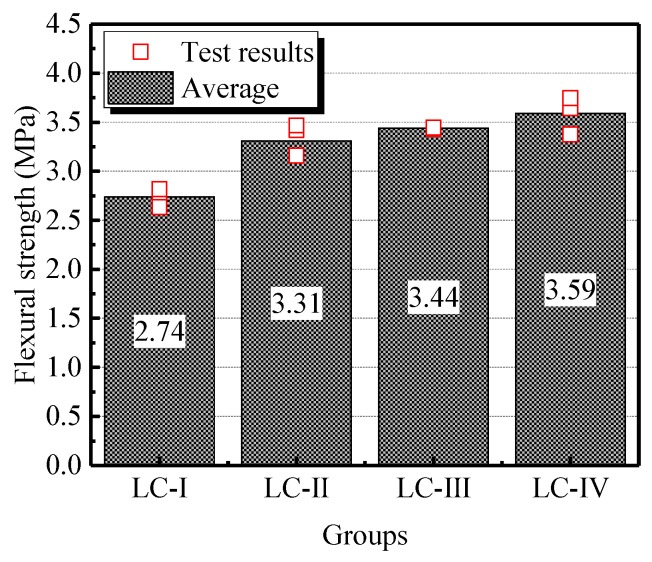
Flexural strength of specimens in four groups.

Figure 4 shows the flexural strength of each specimen and the average values of the four groups. For the specimens of LC-Ⅰ, LC-Ⅱ and LC-Ⅲ, the flexural strength was enhanced with the increasing size of LWA, and the maximum average flexural strength of LC-Ⅲ was 31% higher than the minimum one of LC-Ⅰ. The specimens of LC-Ⅳ exhibited the highest flexural strength of all groups of LWA, which is 3.59 MPa on average.

In the past, the increasing flexural strength was attributed to the better bond performance between the aggregate and mortar matrix adding enhanced homogeneity provided by the lower size of aggregate [19,20]. For LC, ITZ is no longer the weakest part when subjected to tensile stress, while LWA will firstly fracture; therefore, in line with the test results of splitting tensile strength, except for LC-Ⅱ, the flexural strength of specimens give support to the conclusion that the larger size of aggregate is accompanied with better performance, resulting in the higher flexural strength.

### 3.6. Relations of Splitting Tensile, Flexural, and Compressive Strength

**Figure 5:**
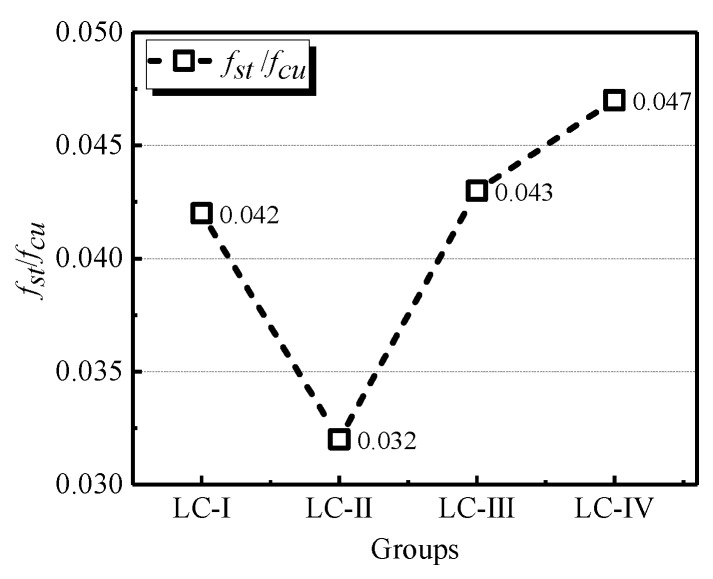
Ratios of the splitting tensile strength to the compressive strength of LC.

**Figure 6:**
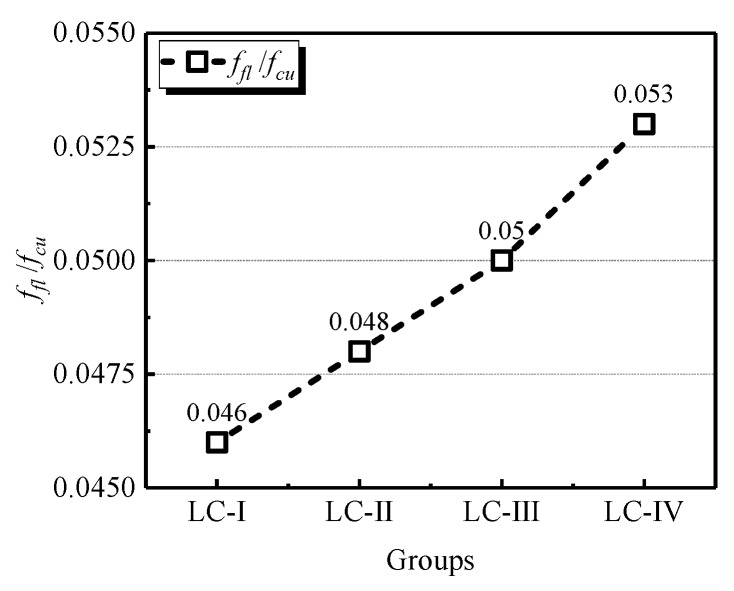
Ratios of the flexural strength to the compressive strength of LC.

Figure 5 and Figure 6 show the splitting tensile-to-28-day compressive strength and the flexural-to- compressive strength ratios, respectively. The splitting strength was found between 3.2% and 4.7% of the compressive strength, and the flexural strength located within the range between 4.6% and 5.3% of the compressive strength. Both splitting tensile strength and flexural strength were smaller than 6% of the cubic compressive strength, which are significantly lower than the values of NWC. This is due to the tensile strength of LC being directly limited by the strength of LWA, and the strength of the used LWA is significantly lower than the normal weight aggregate, which reduces the ratios of that of splitting tensile strength and flexural strength to compressive strength. Furthermore, it was concluded that the variation of LWA size contributed little difference to the discussed conversions, because the alteration of these two ratios with the grading change of aggregate were in line with the variation of splitting tensile strength and flexural strength.

## 4. Estimation Model for 28-day Compressive Strength

### 4.1. Existing Models

Various prediction models have been proposed for the compressive strength of LC, and in the present study, the following equations were adopted to predict the compressive strength of LC.

#### 4.1.1. Weigler and Karl

The expression suggested by CEB-FIP can be written as

(3) $$R_{{}_{28}}=\frac{b_{1}}{w/c}+b_{2}\gamma_{k}+b_{3}V_{Q}+a$$

where

*R*_28_ is the 28-day compressive strength of LC, in MPa;
*w*/*c* is the ratios of water to cement content;
*γ_k_* is the particle density of LWA;
*V_Q_* is the volume fraction of LWA in the concrete mixture;
*b*_1_, *b*_2_, *b*_3_, *a* are parameters determined by experiments.

#### 4.1.2. Beckman and Little

Beckman et al. proposed a prediction of compressive strength by introducing the cylinder compressive strength of LWA, which reads

(4) $$R_{28}=\frac{b_{1}}{w/c}+b_{2}R_{c}+b_{3}R_{T}-a$$

where

*R_c_* is the strength of cement matrix, in MPa;
*R_T_* is the cylinder compressive strength of LWA, in MPa;
*b*_1_, *b*_2_, *b*_3_, *a* are the parameters determined by experiments.

#### 4.1.3. Wang et al.

Taking the strength of LC and the density of concrete, Wang et al. proposed an equation to predict the 28-day compressive strength of LWA, which is shown as

(5) $$\frac{R_{28}-R_{T}}{R_{c}}=a\times \frac{C}{\rho_{d}}+b$$

where

*C* is the cement consumption;
*ρ_d_* is the dry apparent density of LC, in kg/m^3^
*a* and *b* are parameters determined by the experiments.

#### 4.1.4. Validation of Predicted Models

Parameters of three models were obtained by using the test results, and the predicted compressive strengths are listed in Table 6. The predictions of compressive strength showed mean ratios of predicted-to-experimental results from 0.987 to 1.001, with standard deviation (SD) and coefficient of variance (CoV) of 0.029, which verifies the validity of these models. Moreover, from the variation in the parameters of these models, it was clear that not all parameters are sensitive to the change of aggregate size, such as *b*_1_, *a* of Weigler’s equation. Therefore, the choice of the parameter of the prediction model is not necessary to include all types of influencing factors, but the W/C ratio and influence of LWA (including the strength and volume fraction) are required to give rise to better predictions in accordance with the results of the three existing models.

### 4.2. Proposed Model

On the basis of existing prediction models, a simple estimation model of the 28-day compressive strength of LC was proposed, combining with the effect of aggregate size.

#### 4.2.1. Composition of LC

Considering the denser ITZ of LC compared to that of NWC, various researches regarded the LC as a two-phase material consisting of LWA and mortar matrix rather than a three-phase material possessing aggregate, mortar matrix and ITZ [24,25]. Accordingly, the establishment of a mechanical model for the compressive strength of LC was taking the LC as composites of LWA and the mortar matrix in the following sections.

#### 4.2.2. Basic Mechanical Model

**Figure 7:**
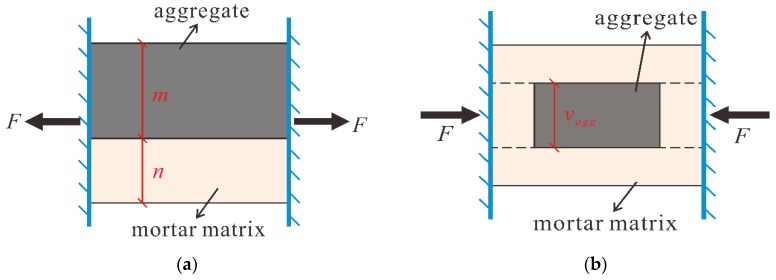
Equivalent model and the modification: (**a**) Existing mechanical model; (**b**) Modification of existing model.

The equivalent method proposed by Du et al. was introduced to simply develop a mechanical model for the compressive strength of the LC [26]. As shown in Figure 7a, the effective properties of two-phase concrete can be illustrated as a parallel computational model of aggregates and mortar matrix. This is definitely true on the basis of the high strength of normal weight aggregate; nonetheless, and some low-strength LWAs usually initiate the fracture or the cracking behavior of LC. To reflect the weak feature of LWA, a modification was made on the existing parallel computational model, which is given Figure 7b. At the inner of LC, the relation between aggregate and surrounding matrix can be assumed as a series connection when subjected to stress, and the rest of the mortar matrix is still regarded to be parallel to this part.

In the parallel computational model for two-phase concrete, the strength of concrete was determined by the maximum value of the strengths of aggregate and the mortar matrix. For the LC, the propagation of micro cracks in LWA prior to the failure of concrete decreased the effective area of the bearing surface. Therefore, parameters for describing the volumetric effect of LWA and the mortar matrix were introduced into the estimation model, which are related to the real volume fraction. Additionally, according to the test results in previous sections, the volumetric parameters should be amended considering the effect of aggregate size.

Concluding the description in former sections, the calculation for the compressive strength of the LC reads:

(6) $$f_{c}^{es}=\beta \max\left[v_{agg}f_{agg},v_{mo}f_{mo}\right]$$

where

*f_c_^es^* is the estimated compressive strength of LC;
*v_agg_* is the volumetric parameter for LWA;
*f_agg_* is the cylinder compressive strength of LWA;
*v_mo_* is the volumetric parameter for mortar matrix;
*β* is a factor considering the effect of aggregate size;
*f_mo_* = the strength of the mortar matrix, which can be estimated by 11.30×(W/C)^−1.713^ [27], where W/C was taken as the W/B ratio in this present study.

#### 4.2.3. Determination of Volumetric Parameter

According to the 28-day compressive strength obtained in the tests, the parameter, *v_mo_*, was determined, by which the effect of aggregate size on the compressive strength of LC can be then reflected. The results of *v_mo_* for the 28-day compressive strength of four groups of specimens are given in Table 7.

As given in Table 7, the *v_mo_* of the four groups of LC were generally closed to 0.6, hereinto the greatest deviation was 8.3%, which confirms that *v_mo_* was related to the volume fraction of binder materials in mixtures, considering the 60% of binder materials for four groups of LC by volume. Moreover, from the view of volume fraction, the varying *v_mo_* indicates that the grading of LWA virtually affected the effectiveness of mortar strength by influencing the quality of compaction or enhancing the ITZ through internal curing mechanism.

#### 4.2.4. Comparison with Test Results

To evaluate the adaptability of the proposed estimation model, the compressive strengths of plain LC obtained by a previous work conducted by the authors, were used to make comparisons [8]. Owing to the relatively higher content of medium size LWA, the *β* was empirically taken as the interception value between 1.08 and 1.05 as 1.065. The details of collected compressive strength and the estimated results are given in Table 8. The proposed model gave rise to the experimental-to-predicted compressive strength ratio of 1.15 with standard deviation of 0.13. It is clear that the accuracy of estimation increased with the decrease in W/B. Therefore, the proposed estimating method was recommended for the LC having W/B lower than 0.34. Further relevant work should be conducted on the strength of the mortar matrix affected by the ITZ of the LC to improve the accuracy.

## 5. Conclusions

Effects of aggregate size on the density, compressive strength, splitting tensile strength and flexural strength of LC were investigated by conducting a series of experiments, and then, an estimation model for the compressive strength of LC was established. According to the results of investigations, the following conclusions can be drawn:

(1) The absence of medium-sized particles decreased the compaction of LC, therefore the density and compressive strength was negatively affected. Specimens having medium-sized LWA showed the highest compressive strength up to 95 MPa.
(2) Compared with the results of low-quality LWA in literatures that lower aggregate size leads to higher strength of LWAC [10], the excessively lower size of expanded shale LWA undermined both the compaction, splitting tensile and flexural strength of LC, while a rational gradation of LWA was beneficial to the splitting tensile and flexural strength.
(3) The splitting tensile strength and flexural strength of tested LC was located between 3.2% to 4.7% and 4.6% to 5.3% of compressive strength, respectively, which are lower than NWC. The change of aggregate size hardly influences these portions.
(4) Validation of three existing strength models was confirmed, and by analyzing the required parameters, an estimation model for LC was established. The estimating predictions were compared with the test results. The accuracy of the proposed model increased with the decrease in W/B ratio, therefore the model was recommended to be used for the LC having W/B ratio lower than 0.34.

## Figures and Tables

**Table 1:** Chemical compositions and physical properties of Portland ordinary cement.

Chemical Composition	Ordinary Portland Cement (%)
Calcium Oxide (CaO)	62.81
Silicon Dioxide (SiO_2_)	20.36
Aluminum Oxide (Al_2_O_3_)	5.67
Ferric Oxide (Fe_2_O_3_)	3.84
Magnesium Oxide (MgO)	2.68
Sulfur Trioxide (SO_3_)	2.51
K_2_O	0.87
Na_2_O	0.19
Loss on Ignition	1.07
Specific Gravity (g/cm^3^)	3.14
Fineness (m^2^/kg)	329

**Table 2:** Cumulative passing for four aggregate grades.

Sieve Size (mm)	Cumulative Passing (%)			
	Grade Ⅰ	Grade Ⅱ	Grade Ⅲ	Grade Ⅳ
16	100	100	100	100
9.5	100	100	0	61
4.75	100	0	0	31

**Table 3:** Mixture of lightweight concrete (LC) (per m^3^).

	Cement (kg)	FA (kg)	SF (kg)	SP (kg)	Water (kg)
1 m^3^	440	66	44	4.2	148.5

**Table 4:** Details of the tests and specimens.

Test	Dimensions (mm^3^)	Number of Specimens (Group × No. in Each Group)
Compressive Strength Test	100 × 100 × 100	4 × 15
Splitting Tensile Strength Test	100 × 100 × 100	4 × 3
Flexural Strength Test	100 × 100 × 400	4 × 3

**Table 5:** Test results of specimens.

groups	*ρ_d_* (kg/m^3^)	*f_cu_* (MPa)					*f_st_* (MPa)	*f_fl_* (MPa)
		3 d	7 d	28 d	90 d	120 d		
LC60-Ⅰ	1870	31.5	37.0	61.5	68.1	67.9	2.47	2.78
		32.2	45.0	58.1	70.4	82.0	2.48	2.63
		32.9	40.7	60.5	81.1	77.1^b^	2.61	2.82
LC60-Ⅱ	1910	43.8	54.5	72.1	95.5	N/A	N/A	3.42
		45.7	55.9	66.5	91.0	N/A	2.25	3.47
		45.4	52.8	69.1	92.9	N/A	2.12	3.16
LC60-Ⅲ	1902	43.3	52.6	67.5	90.2	84.7	2.94	3.43
		39.9	52.6	71.4	72.1^b^	84.7	3.00	3.44
		43.5	49.2	67.6	67.6	89.8	N/A	N/A
LC60-Ⅳ	1911	45.5	53.1	69.5	69.5	72.4	3.15	3.64
		37.4	50.8	67.4	82.8	86.0	3.07	3.38
		41.7	35.3*^a^*	65.1	54.6*^a^*	73.1	3.35	3.75

*^a^*: the data cannot be used to analyzed owing to the error in casting process; ^b^: these values were chosen as representative values instead of the average values in one group; N/A: the test was interrupted prior to the maximum strength owing to uncontrollable factors.

**Table 6:** Parameters and predicted compressive strengths of existing models.

Groups	Weigler and Karl [21]					Beckman and Little [22]					Wang et al. [23]		
	*b*_1_	*b*_2_	*b*_3_	*a*	*f_c_*	*b*_1_	*b*_2_	*b*_3_	*a*	*f_c_*	*a*	*b*	*f_c_*
LC60-Ⅰ	0.580	0.037	2.173	1.474	60.49	0.598	0.474	0.966	−1.480	60.82	0.000160	0.499	60.03
LC60-Ⅱ	0.580	0.043	2.273	1.474	69.71	0.601	0.560	0.990	−1.480	70.16	0.000184	0.586	69.24
LC60-Ⅲ	0.580	0.043	2.214	1.474	69.29	0.601	0.556	0.979	−1.480	69.66	0.000184	0.582	68.83
LC60-Ⅳ	0.580	0.042	2.213	1.474	67.81	0.601	0.543	0.977	−1.480	68.19	0.000178	0.568	67.36
Mean	0.993					0.987					1.001		
SD	0.029					0.029					0.029		
CoV	0.029					0.029					0.029		

**Table 7:** The values of volumetric parameter and the estimated 28-day compressive strength.

Groups	*f_mo,28d_* (MPa)	*β*	Predicted *f_cu_* (MPa)	SD
LC-Ⅰ	106.45	0.93	59.61	0.0167
LC-Ⅱ		1.08	69.19	0.0264
LC-Ⅲ		1.08	68.13	0.0208
LC-Ⅳ		1.05	65.80	0.0206

**Table 8:** Comparison of test and estimated results.

Strength Grade	W/B	*f_cu_* (MPa)	*β*	Predicted *f_mo_* (MPa)	Predicted *f_cu_* (MPa)	Experimental-to-Predicted
**LC40**	0.4	47.2	1.07	54.29	34.69	1.36
	0.38	47.9		59.28	37.88	1.26
**LC50**	0.36	52.7		65.03	41.55	1.27
	0.34	55.7		71.72	45.83	1.22
	0.32	56.6		79.57	50.85	1.11
	0.3	58.1		88.87	56.79	1.02
**LC60**	0.28	64.8		100.02	63.91	1.01
	0.27	68.8		106.45	68.02	1.01
	0.26	76.7		113.56	72.56	1.06
**Mean**						1.15
**SD**						0.13
